# Prospective comparison of ^18^F-PSMA-1007 PET/CT, whole-body MRI and CT in primary nodal staging of unfavourable intermediate- and high-risk prostate cancer

**DOI:** 10.1007/s00259-021-05296-1

**Published:** 2021-03-13

**Authors:** Simona Malaspina, Mikael Anttinen, Pekka Taimen, Ivan Jambor, Minna Sandell, Irina Rinta-Kiikka, Sami Kajander, Jukka Schildt, Ekaterina Saukko, Tommi Noponen, Jani Saunavaara, Peter B. Dean, Roberto Blanco Sequeiros, Hannu J. Aronen, Jukka Kemppainen, Marko Seppänen, Peter J. Boström, Otto Ettala

**Affiliations:** 1 Turku PET Centre, University of Turku and Turku University Hospital, Turku, Finland; 2 Department of Urology, University of Turku and Turku University Hospital, Turku, Finland; 3 Institute of Biomedicine and Department of Pathology, University of Turku and Turku University Hospital, Turku, Finland; 4 Department of Radiology, Icahn School of Medicine at Mount Sinai, New York, NY USA; 5 Department of Diagnostic Radiology, University of Turku and Turku University Hospital, Turku, Finland; 6 Department of Radiology, Tampere University Hospital, Tampere, Finland; 7 Department of Clinical Physiology and Nuclear Medicine, Helsinki University Central Hospital, Helsinki, Finland; 8 Department of Medical Physics and Nuclear Medicine, University of Turku and Turku University Hospital, Turku, Finland; 9 Department of Clinical Physiology, Nuclear Medicine and Turku PET Centre, University of Turku and Turku University Hospital, Turku, Finland

**Keywords:** Prostate cancer, Primary staging, Lymph node metastasis, ^18^F-PSMA-1007 PET/CT, WBMRI, CT

## Abstract

**Purpose:**

To prospectively compare ^18^F-prostate-specific membrane antigen (PSMA)-1007 positron emission tomography (PET)/CT, whole-body magnetic resonance imaging (WBMRI) including diffusion-weighted imaging (DWI) and standard computed tomography (CT), in primary nodal staging of prostate cancer (PCa).

**Methods:**

Men with newly diagnosed unfavourable intermediate- or high-risk PCa prospectively underwent ^18^F-PSMA-1007 PET/CT, WBMRI with DWI and contrast-enhanced CT within a median of 8 days. Six readers (two for each modality) independently reported pelvic lymph nodes as malignant, equivocal or benign while blinded to the other imaging modalities. Sensitivity, specificity and accuracy were reported according to optimistic (equivocal lesions interpreted as benign) and pessimistic (equivocal lesions interpreted as malignant) analyses. The reference standard diagnosis was based on multidisciplinary consensus meetings where available histopathology, clinical and follow-up data were used.

**Results:**

Seventy-nine patients completed all the imaging modalities, except for one case of interrupted WBMRI. Thirty-one (39%) patients had pelvic lymph node metastases, which were detected in 27/31 (87%), 14/31 (45%) and 8/31 (26%) patients by ^18^F-PSMA-1007 PET/CT, WBMRI with DWI and CT, respectively (optimistic analysis). In 8/31 (26%) patients, only ^18^F-PSMA-1007 PET/CT detected malignant lymph nodes, while the other two imaging modalities were reported as negative. At the patient level, sensitivity and specificity values for ^18^F-PSMA-1007 PET/CT, WBMRI with DWI and CT in optimistic analysis were 0.87 (95%CI 0.71–0.95) and 0.98 (95%CI 0.89–1.00), 0.37 (95%CI 0.22–0.55) and 0.98 (95%CI 0.89–1.00) and 0.26 (95%CI 0.14–0.43) and 1.00 (95%CI 0.93–1.00), respectively.

**Conclusion:**

^18^F-PSMA-1007 PET/CT showed significantly greater sensitivity in nodal staging of primary PCa than did WBMRI with DWI or CT, while maintaining high specificity.

**Clinical trial registration:**

Clinicaltrials.gov ID: NCT03537391

**Supplementary Information:**

The online version contains supplementary material available at 10.1007/s00259-021-05296-1.

## Introduction

The presence of pelvic lymph node metastases at initial staging is an important prognostic factor in primary prostate cancer (PCa) [1]. Following radical treatment of localized PCa, such as prostatectomy or external beam radiotherapy, some men are diagnosed with nodal recurrence [2]. This can be partly attributed to the inability of conventional imaging methods to correctly stage patients at the time of initial diagnosis. A more accurate determination of the initial extent of the disease using next-generation imaging modalities could improve therapeutic planning and possibly treatment outcome [3].

Abdominopelvic imaging with conventional computed tomography (CT) is still recommended in primary nodal staging of PCa, although the sensitivity of CT in detecting lymph node metastases is modest [4]. Pelvic magnetic resonance imaging (MRI) with diffusion-weighted imaging (DWI) is the method of choice for assessing local tumour extent, and it plays an important role in the detection of regional lymph node metastases [5]. Moreover, determining the overall extent of PCa with whole-body MRI (WBMRI) has gained increasing interest [6] .

Prostate-specific membrane antigen (PSMA) positron emission tomography (PET) imaging has recently been introduced in PCa imaging [7]. ﻿ Accumulating evidence supports the use of PSMA PET/CT for the restaging of PCa after biochemical recurrence. However, there is less evidence supporting its use in primary staging, and yet the data are mainly limited to ^68^Ga-labelled PSMA tracers. Alternatively, novel ^18^F-labelled PSMA ligands, such as ^18^F-PSMA-1007 [8], are able to offer longer half-life, superior energy characteristics and higher image resolution compared with ^68^Ga-labelled tracers. In addition, ^18^F-PSMA-1007 is only minimally excreted by the urinary tract, an advantage in pelvic imaging. There is preliminary evidence that ^18^F-labelled PSMA tracers might have a higher incidence of benign uptake in bone tissue and unspecific lymph nodes [9, 10], although no prospective comparative studies with ^68^Ga-labelled tracer are available.

To date, only a limited number of studies evaluating ^18^F-labelled PSMA tracers in the detection of PCa regional lymph node metastases have been published [11–13].

We have previously prospectively compared the diagnostic performance of next-generation (^18^F-PSMA-1007 PET/CT, WBMRI with DWI and SPECT/CT) and conventional imaging modalities (CT and bone scintigraphy) in primary distant metastasis staging of PCa [10].

Using the same patient cohort, the aim of the current study was to prospectively compare ^18^F-PSMA-1007 PET/CT, WBMRI using DWI and CT in primary nodal staging of men with unfavourable intermediate- and high-risk PCa.

## Material and methods

### Study design and patient population

This is a prospective non-randomized registered (NCT03537391) single-centre trial that included patients with newly diagnosed histologically confirmed unfavourable intermediate- or high-risk PCa ﻿(International Society of Urological Pathology grade group ≥3 and/or prostate-specific antigen [PSA] ≥20 and/or cT ≥ T3). Exclusion criteria included any previous PCa imaging for metastasis staging, PCa treatment before enrolment and contraindications for MRI. Administration of androgen deprivation therapy (ADT) at enrolment was permitted if necessary for symptomatic very high-risk PCa patients. All participants underwent ^99m^Tc-HMDP planar bone scintigraphy, ^99m^Tc-HMDP SPECT/CT, contrast-enhanced abdominopelvic and thoracic CT, WBMRI with DWI and ^18^F-PSMA-1007 PET/CT within 2 weeks of enrolment and without a prespecified sequence. Since the current study solely focused on regional nodal staging, the following imaging modalities were evaluated:

1. Standard imaging: contrast-enhanced CT
2. Imaging under evaluation: WBMRI with DWI and ^18^F-PSMA-1007 PET/CT

### Imaging modalities

#### Contrast-enhanced CT

Abdominopelvic and thoracic CT was performed with a Discovery NM/CT 670 CZT, a digital SPECT/CT imaging system, including Optima CT540 subsystem (GE Healthcare, Tirat, Hacarmel, Israel). A helical CT tomogram with a modulated mAs (noise index ~30), a rotation time of 0.5 s, 120 kVp, a pitch of 0.938 and 1.25-mm slice thickness was acquired. Soft tissue, bone and lung kernels were employed with a 40% dose reduction in the Adaptive Statistical Iterative Reconstruction (ASIR, GE Healthcare, USA) algorithm. A biphasic contrast-enhanced CT protocol (arterial phase of 10 s, followed by venous phase at 30 s) was performed. Contrast agent (Omnipaque (iohexol)™ GE Healthcare, iodine concentration of 350 mg/ml) was used unless clinical contraindications were present.

#### Whole-body MRI

WBMRI imaging was performed using a Siemens Magnetom Avanto fit 1.5 T MR system (Siemens Healthcare GmbH, Erlangen, Germany). WBMRI acquisition protocol consisted of axial T2-weighted fat suppressed (FS) half-Fourier single shot turbo-spin echo images (HASTE), axial short-tau inversion recovery (STIR) DWI, *b*-values 0, 50, 900 s/mm^2^ and coronal 3D T1-weighted volumetric interpolated breath-hold examination (VIBE) Dixon sequences. In addition, whole spine sagittal T1- and T2-weighted STIR turbo spin-echo (TSE) sequences and axial STIR DWI images from the level of the pelvis, b values 0, 1500 s/mm^2^, were acquired.

#### ^18^F-PSMA 1007 tracer synthesis and PET/CT

^18^F-PSMA-1007 tracer was manufactured by MAP Medical Technologies Oy, Curium Pharma (Helsinki, Finland), as previously described [14].

The PET/CT study was carried out with Discovery MI digital PET/CT system (GE Healthcare, Milwaukee, WI, USA), with a 128 slice CT and a 3D PET imaging capability. The PET imaging field of view (FOV) was 70 cm in diameter and 20 cm in axial length. Transmission scan for attenuation correction was performed using a low-dose (noise index 30, automatic 3D current modulation, 10–120 mAs and 120 kVp) CT protocol. A static emission scan was acquired from vertex to mid-thigh (6 bed positions, 2 min/bed). The sinogram data were corrected for deadtime, decay and photon attenuation and reconstructed in a 256 × 256 matrix. Image reconstruction utilized a Q. Clear method (a Bayesian penalized likelihood reconstruction algorithm for PET) with β value of 500 incorporating random and scatter corrections. The final in-plane FWHM (full-width half-maximum) of the systems is <5 mm.

### Imaging interpretation and reference standard

There were a total of six readers (4 radiologists and 2 nuclear medicine physicians), two for each of the three imaging modalities. Each imaging modality was independently reviewed by the same pair of experienced modality-based experts, blinded for the other modalities.

The pelvic lymph nodes were reported as malignant, equivocal or benign, and these data were collected on an electronic database [15]. Both optimistic (equivocal lesions interpreted as benign) and pessimistic (equivocal lesions interpreted as malignant) analyses were performed to resolve equivocal lesion status. Lesions were interpreted in all modalities according to clinical expertise and following current guidelines [3, 16]. In CT and WBMRI, lymph node diameter (short diameter > 8 mm) and morphology (rounded) were used to determine malignancy. In MRI, diffusion restriction was also used to assess nodal invasion, especially in normal-sized lymph nodes. In ^18^F-PSMA-1007 PET/CT, lymph nodes in a typical site of PCa metastasis and with tracer uptake (expressed as standardized uptake value [SUVmax]) above the blood pool were considered malignant. Imaging studies were interpreted using Advantage Workstation (version 4.7, GE Healthcare, Buc, France), Weasis Medical Viewer (version 3.5.3, University Hospital of Geneva, Switzerland) and Vue PACS (version 12.2.0.1007, Carestream Health Inc., Rochester, USA).

For the validation of all reported lesions, the reference standard diagnosis was utilized, which included histopathological specimens (when available), information from all primary imaging modalities, follow-up imaging and clinical follow-up data. When histopathology was not available, lymph nodes were considered malignant when at least three of the following criteria were met: (1) concordance between primary imaging modalities, (2)﻿ increase in size or number of lymph nodes during follow-up imaging, (3) decrease in size or number of lymph nodes during follow-up imaging in response to treatment, (4) increase in serum PSA suggesting progression, (5) decrease in serum PSA in response to treatment, (6) increase in PSMA uptake during follow-up imaging (when available), and (7) decrease in PSMA uptake during follow-up imaging (when available) in response to treatment.

The reference standard diagnosis was determined at the lesion level in a regularly organized consensus meeting by a multidisciplinary team including two urologists, one uropathologist, two radiologists (CT and MRI specialists) and two nuclear medicine physicians.

### Histopathological analysis

Surgical tissue specimens from pelvic lymph node dissection (PLND, i.e., removal of lymphatic tissue around external and internal iliac vessels and obturator nerve starting from the ureter crossing and extending to the pelvic wall) were fixed in 10% buffered formalin for minimum of 24 h. The number of palpable lymph nodes identified on each side was determined, and the lymph nodes were cut in 3–4 mm sections before routine tissue processing. Consecutive histological sections of 4 μm thickness were used for haematoxylin and eosin (H&E) staining and for immunohistochemistry. Epitope unmasking was done by microwaving the slides in Tris-EDTA buffer. PSMA staining was carried out with a Lab Vision autostainer (Thermo Fisher Scientific) using a mouse monoclonal PSMA antibody (Dako, clone M3620, 1:100) and Envision detection kit (EP192). Pan-cytokeratin staining was carried out using BenchMark ULTRA automated slide stainer (Ventana Medical Systems, Tucson, Arizona, USA) and anti-pan-cytokeratin antibody (clone AE1/AE3/PCK26, 46.3 μg/ml). All the histological slides were reviewed by one board-certified experienced uropathologist blinded to the imaging modality results.

### Statistical analysis

The sample size estimation for this clinical trial has been previously described [10]. Descriptive statistics including median, interquartile range (IQR) and range were used. Sensitivity, specificity and accuracy were reported with 95% confidence interval (CI) and compared between modalities with Fisher’s exact test. For both patient- and lesion-based statistical analysis, correct side of the pelvis (right or left) was considered to achieve correct agreement with the reference standard diagnosis. The inter-reader agreement was assessed using Cohen’s Kappa (95% CI). Pearson’s *r* was used to study correlation between PSMA SUVmax and lesion’s size. *p*-values less than 0.05 were considered statistically significant. Statistical analyses were performed with JMP® System, version 14.2.0 for MacOS (SAS Institute Inc., Cary, NC, USA).

## Results

Seventy-nine patients were included in this study. All patients underwent all imaging modalities except for one case of interrupted WBMRI due to unexpected claustrophobia. Patient characteristics are summarized in Table 1. The median age was 72 years (interquartile range [IQR] 66–77; range 52–87), and median PSA was 12 (IQR 7–23; range 3–2000). The median interval between the first and last imaging was 8 days (IQR 6–12; range 1–44). The median administered activity of ^18^F-PSMA-1007 was 250 MBq (IQR 246–256; range 206–279), and PET/CT scan was acquired at a median of 60 min (IQR 60–60; range 59–63) from tracer injection. The median follow-up period was 21 months (IQR 19–25; range 16–29). Five patients received ADT therapy at enrolment due to symptomatic very high-risk PCa. In all of these patients, metastatic disease was detected, and all imaging modalities were performed within 3 weeks from enrolment. In particular, the median interval between the initiation of ADT and ^18^F-PSMA PET/CT was 7 days (IQR 3–17; range 2–22).

**Table 1:** Patient demographics

Age	Median ([IQR] range)
Years	72 ([66–77] 52–87)
PSA	Median ([IQR] range)
ng/ml	12 ([7–23] 3–2000)
Clinical T-category ^a^	*n* (%)
cT1	7 (11)
cT2	37 (46)
cT3	27 (33)
cT4	8 (10)
Biopsy GGG	*n* (%)
1^b^	3 (4)
2	1 (1)
3	29 (37)
4	13 (16)
5	33 (42)
Primary treatment^c^	*n* (%)
RALP	5 (6)
RALP + PLND	17 (22)
EBRT	37 (47)
TULSA	2 (3)
ADT	17 (21)
Watchful waiting	1 (1)

*PSA* prostate-specific antigen, *GGG* Gleason grade group, *RALP* robot-assisted laparoscopic prostatectomy, *PLND* pelvic lymph node dissection, *EBRT* external beam radiotherapy with (*n* = 36) or without (*n* = 1) *ADT* androgen deprivation therapy, *TULSA* transurethral ultrasound ablation of prostate; ADT with (*n* = 4) or without (*n* = 13) early chemotherapy with docetaxel

^a^Clinical T-category was determined based on transrectal ultrasound and digital rectal examination before any imaging

^b^All patients with GGG 1 had PSA >20 ng/ml

^c^All treatments were performed and/or initiated after the imaging studies, except for 5 patients, who began ADT at enrolment due to symptomatic very high-risk PCa. ﻿In two cases, palliative transurethral resection of the prostate was performed due to bladder outlet obstruction prior to EBRT, and one case underwent palliative TULSA combined with ADT

According to EAU risk group classification, 17/79 (22%) patients belonged to unfavourable intermediate- and the rest to high-risk group (62/79, 78%). Consensus staging results after all imaging reports were as follows: 41 patients had localized disease, 18 had locally advanced disease, and 20 had distant metastatic disease.

Thirty-one (39%) patients were deemed to have pelvic lymph node metastatic disease, which, in optimistic analysis, was detected in 27/31(87%), 14/31(45%) and 8/31 (26%) patients by ^18^F-PSMA-1007 PET/CT, WBMRI with DWI and CT, respectively. In 8/31 (26%) patients, only ^18^F-PSMA-1007 PET/CT was able to detect metastatic lymph nodes, while the other two imaging modalities were reported as negative. Sensitivity, specificity and accuracy values at the patient-level are given in Table 2. ^18^F-PSMA-1007 PET/CT significantly outperformed WBMRI with DWI and CT in sensitivity and accuracy. Inter-reader agreement for ^18^F-PSMA-1007 PET/CT at the patient-level was superior compared to WBMRI with DWI and CT, with Kappa values of 0.89, 0.47 and 0.69, respectively, in optimistic analysis (Supplementary Table S1).

**Table 2:** Sensitivity, specificity and accuracy of both readers of each imaging modality in pessimistic and optimistic analysis at the patient level

Imaging modality	Reader	Pessimistic analysis			Optimistic analysis		
		Sensitivity (95%CI)	Specificity (95%CI)	Accuracy (95%CI)	Sensitivity (95%CI)	Specificity (95%CI)	Accuracy (95%CI)
CT	1	0.39 (0.24–0.56) ^a,b^	0.94 (0.83–0.98)	0.72 (0.61–0.80) ^a,b^	0.16 (0.07–0.32) ^a,b^	1.00 (0.93–1.00)	0.67 (0.56–0.76) ^a,b^
	2	0.32 (0.19–0.50) ^a,b^	0.94 (0.83–0.98)	0.70 (0.59–0.79) ^a,b^	0.26 (0.14–0.43) ^a,b^	0.98 (0.89–0.99)	0.70 (0.59–0.79) ^a,b^
WBMRI with DWI	1	0.40 (0.25–0.58) ^a,b^	0.96 (0.86–0.99)	0.74 (0.63–0.83) ^a,b^	0.37 (0.22–0.55) ^a,b^	0.98 (0.89–1.00)	0.74 (0.63–0.83) ^a,b^
	2	0.50 (0.33–0.67) ^a,b^	0.91 (0.80–0.97)	0.75 (0.65–0.84) ^a,b^	0.37 (0.22–0.55) ^a,b^	0.98 (0.89–1.00)	0.74 (0.63–0.83) ^a,b^
^18^F-PSMA-1007 PET/CT	1	0.84 (0.67–0.93)	0.96 (0.86–0.99)	0.91 (0.83–0.96)	0.77 (0.60–0.89)	0.98 (0.89–1.00)	0.90 (0.81–0.95)
	2	0.90 (0.75–0.97)	0.94 (0.83–0.98)	0.93 (0.85–0.96)	0.87 (0.71–0.95)	0.96 (0.86–0.99)	0.92 (0.84–0.96)

*CT* computed tomography, *WBMRI* whole-body magnetic resonance imaging, *DWI* diffusion-weighted imaging, ^*18*^*F-PSMA-1007 PET/CT* prostate-specific membrane antigen positron emission tomography-CT, *CI* confidence interval

^a^Statistically significant difference (*p* < 0.05) compared to ^18^F-PSMA-1007 PET/CT reader 1

^b^Statistically significant difference (p < 0.05) compared to ^18^F-PSMA-1007 PET/CT reader 2

At the lesion level, 206 lymph nodes were interpreted as malignant (the reference standard diagnosis). The number of true positive, false positive and false negative lesions for each imaging modality and reader is shown in Table 3. The detection rate of lymph node metastases for ^18^F-PSMA-1007 PET/CT was 83%, compared to 58% for WBMRI with DWI and 52% for CT. Out of all the metastatic lesions detected by ^18^F-PSMA 1007 PET/CT, 126/170 (74%) were smaller than the anatomical cutoff value of 8 mm, of which 90 lymph nodes had the short diameter between 5 and 8 mm and 36 lymph nodes <5 mm (Supplementary Fig. S1). SUVmax intensity did not show correlation with lymph node’s dimensions (*r*^2^ = 0.010; *p* = 0.33).

**Table 3:** The total number of reported lesions by both readers of each imaging modality and their concordance with the reference standard diagnosis at the lesion level

Imaging modality	Reader	Number of positive lesions reported	Number of true positive lesions	Detection rate of true positive lesions	Number of false positive lesions	Number of false negative lesions	Number of equivocal lesions reported
CT	1	52	52	25%	0	154	36
	2	146	107	52%	39	99	12
WBMRI with DWI	1	93	91	44%	2	110	1
	2	179	120	58%	59	81	9
^18^F-PSMA-1007 PET/CT	1	178	170	83%	8	36	4
	2	156	144	70%	12	62	1

*CT* computed tomography, *WBMRI* whole-body magnetic resonance imaging, *DWI* diffusion-weighted imaging, ^*18*^*F-PSMA-1007 PET/CT* prostate-specific membrane antigen positron emission tomography-CT

There were 206 lymph node metastases according to reference standard diagnosis

**Fig. 1:**
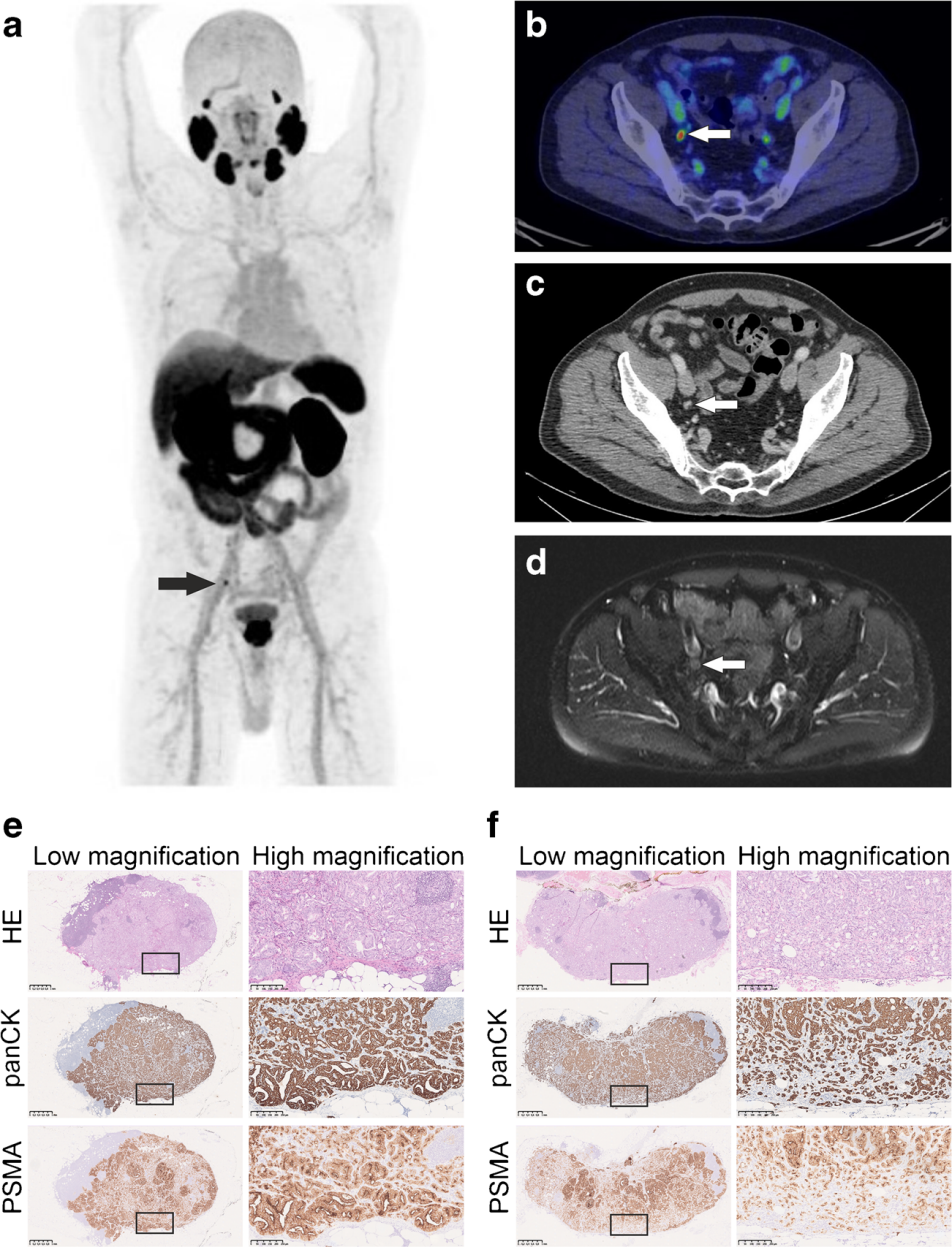
Imaging and histopathological findings of patient 48. ^18^F-PSMA-1007 PET/CT clearly identified one pelvic lymph node metastasis (short diameter: 6 mm) on the right (**a**–**b**) which was retrospectively identified by CT (**c**) and WBMRI (**d**). Histopathological examination confirmed one lymph node metastasis on the right (maximum diameter 7 mm) with intense PSMA and pan-cytokeratin staining in immunohistochemistry (E). On the other hand, ^18^F-PSMA-1007 PET/CT did not detect another lymph node metastasis found in histopathological examination on the left (maximum diameter: 8,5 mm, **f**). This lymph node showed less intense immunohistochemical PSMA staining when compared to one on the right. Boxed areas in low magnification images are shown in high magnification images

Seventeen patients (22%) were treated with robot-assisted laparoscopic prostatectomy (RALP) and PLND, and five of these patients showed lymph node metastases in histopathological examination. The results of the patient- and lesion-based analyses from all the operated patients are shown in Supplementary Table S2 and S3. Compared to the other two imaging modalities, ^18^F-PSMA-1007 PET/CT demonstrated superior sensitivity and accuracy at the patient level (0.67 and 0.82 in optimistic analysis) and the highest detection rate at the lesion level (27%). ^18^F-PSMA-1007 PET/CT was concordant with histopathology in 14/17 (82%) patients, while the corresponding numbers for WBMRI with DWI and CT were 12/17 (71%) and 11/17 (65%), respectively. The number of metastases detected by histopathology and ^18^F-PSMA-1007 PET/CT are presented in Supplementary Table S4. Of the five patients with histologically confirmed lymph node metastases, three were detected by ^18^F-PSMA-1007 PET/CT, while both CT and WBMRI were negative in all five cases. In each of these three patients, additional histologically confirmed lymph nodes metastases were also found, which were not detected by ^18^F-PSMA-1007 PET/CT (Fig. 1). Supplementary Fig. S2 demonstrates one of the two histologically confirmed ^18^F-PSMA-1007 PET/CT false negative cases. The only false positive ^18^F-PSMA-1007 PET/CT case is shown in Supplementary Fig. S3. All the lymph node metastases detected by H&E staining (*n* = 11) were also positive in the immunohistochemical pan-cytokeratin and PSMA staining. Immunohistochemical staining alone revealed additional lymph node micrometastases (*n* = 3) in three patients, each of whom was already diagnosed with other histologically confirmed metastases, thus not affecting the overall nodal status.

Pelvic follow-up imaging was available for 56/62 (90%) of patients not treated with surgery (*n* = 62), including CT (*n* = 15), MRI with DWI (*n* = 31) or ^18^F-PSMA-1007 PET/CT (*n* = 10).

## Discussion

This prospective clinical trial compared standard (CT) and next-generation imaging modalities (PSMA PET/CT using the novel tracer ^18^F-PSMA-1007 and WBMRI with DWI) in primary nodal staging of men with unfavourable intermediate- and high-risk prostate cancer. Thirty-one patients had pelvic lymph node metastases, of which ^18^F-PSMA 1007 PET/CT detected 87%, while the detection rates for WBMRI and CT were 45% and 26%, respectively.^18^F-PSMA-1007 PET/CT showed the highest sensitivity, accuracy and inter-reader agreement.

Many prospective studies that have used histopathology as a validation have already demonstrated adequate diagnostic performance of PSMA PET/CT in primary nodal staging [12, 13, 17–19]. However, only a small number of prospective multimodality comparative studies are available [20–22], and none conducted using ^18^F-PSMA-1007 tracer. Recently, Hofman et al. [20] demonstrated superior diagnostic accuracy of ^68^Ga-PSMA-11 PET/CT in primary staging of men with high-risk PCa in a randomized study setting, showing significantly higher sensitivity (0.85 vs 0.38) and better specificity (0.98 vs 0.91) compared to conventional imaging. In line with the results of our study, the superiority of ^68^Ga-PSMA PET/CT was also confirmed in the subgroup of patients with pelvic nodal metastases. Similarly, the other two non-randomized prospective comparative studies on smaller patient cohorts [21, 22] showed higher performance, especially in terms of sensitivity, of ^68^Ga-PSMA PET/CT compared to MRI and/or CT.

The higher sensitivity of ^18^F-PSMA-1007 PET/CT is most probably based on the fact that lymph node metastases in PCa are not necessarily present only in enlarged lymph nodes [23, 24]. Our data is consistent with this concept since it demonstrated that 74% of all lymph node metastases detected by ^18^F-PSMA-1007 PET/CT were smaller than the anatomical cutoff value of 8 mm (short diameter) used in CT and MRI.

Recent prospective studies that used histopathology as a reference standard showed results similar to our subgroup analysis of operated patients, especially in terms of specificity of PSMA PET/CT in primary nodal staging (Supplementary Table S5). The sensitivity in our subanalysis was only partially in concordance with the results of those studies, which showed heterogeneous values (0.39–0.64). This could be explained to some extent by differences in patient population or in study methodology.

In our subgroup analysis of patients undergoing pelvic lymph node dissection, ^18^F-PSMA-1007 PET/CT was concordant with histology in 82% of the cases. However, there were histologically confirmed nodal metastases (*n* = 9) with longest diameter of ≤4 mm that were not detected by ^18^F-PSMA-1007 PET/CT (Fig. 1, S2). Given the limits of PET/CT resolution, limited accuracy in detecting very small or micrometastases was expected.

Factors other than anatomical size should also affect the detection rate of lymph node metastases. In this patient cohort, lower SUVmax values were observed in smaller lymph nodes with short diameter < 5 mm (Fig. S1). However, PSMA SUVmax values did not correlate with the anatomical size of the lymph nodes. The varying detectability in PSMA PET might be also due to heterogeneity in PSMA expression, as we observed variable intensity in immunohistochemical PSMA staining between positive metastatic lymph nodes (Fig. 1, Supplementary Fig. S2, S4). Nevertheless, none of the lymph node metastases detected by H&E was negative on PSMA immunostaining, in line with recently published data [22]. Weak PSMA expression was occasionally detected also in non-metastatic tissues, as in the germinal centres of lymphoid follicles as well as in the endothelial cells of medullary sinuses (Supplementary Fig. S4). The former may represent tumour cell–derived PSMA phagocytosed by antigen presenting cells as this finding was more frequently observed among patients with metastatic lymph nodes. Further research is needed in this respect.

We might tentatively speculate that another challenge in ^18^F-PSMA-1007 PET/CT interpretation is the risk of false positive cases in possibly reactive lymph nodes located in the very distal iliac region. We observed only one histologically confirmed PSMA false-positive case (Supplementary Fig. S3), where PSA values dropped <0.006 ng/ml during follow-up after surgery.

The main limitation of this study is the relatively small percentage of patients (22%) with histopathologically verified lymph node status, of whom only 5 patients had lymph node metastases. This could have led to a sub-optimal reference standard and a possible underestimation of the true prevalence of lymph node metastases in the majority of participants.

Since this prospective clinical trial was designed to find the most appropriate imaging modality for the overall (local, nodal and distant) staging of men with newly diagnosed unfavourable intermediate- and high-risk PCa, treatment management followed current clinical practice and surgical treatment was not performed in all patients.

Furthermore, a strength of this study is that all patients were examined within a very short time window by three different imaging modalities (^18^F-PSMA-1007 PET/CT WBMRI with DWI and CT) to support the standard reference diagnosis. Another strength of the study is that all patients had long follow-up times supporting the image-based validation of reference standard diagnosis in lesions lacking histopathological evidence.

A small number of the study patients (*n* = 5) with symptomatic very high-risk PCa began ADT therapy at enrolment, which could be considered a minor limitation. However, despite it has been reported that ADT therapy might influence heterogeneously PSMA uptake [25, 26], short-term treatment is unlikely to have affected the lesion detectability. Moreover, all five patients had metastatic disease detected using each of the imaging modalities.

Another limitation of the study is that the effect of the next-generation modalities on treatment decision-making was not prospectively collected and investigated. Nevertheless, additional randomized evidence is needed to support the oncological benefit of detecting earlier metastatic disease with next-generation imaging.

## Conclusion

This prospective comparative clinical trial showed significantly improved sensitivity and accuracy of ^18^F-PSMA-1007 PET/CT over WBMRI with DWI and CT in the detection of pelvic lymph node metastases in primary unfavourable intermediate- and high-risk PCa, while maintaining high specificity. Additional evidence is needed to confirm the possible clinical benefit of the early detection of lymph node metastases by ^18^F-PSMA-1007 PET/CT.

## Supplementary information

ESM 1 (DOCX 13 kb)

ESM 2 (DOCX 18.8 kb)

ESM 3 (DOCX 14 kb)

ESM 4 (DOCX 17 kb)

ESM 5 (DOCX 14 kb)

Fig. S1 SUVmax values of metastatic lymph nodes (reference standard) divided according to lymph node short diameter. Analysis of Reader 1 (higher detection rate) is presented. (PNG 116 kb)

High Resolution Image (TIFF 164 kb)

Fig. S2 Imaging and histopathological findings of Patient 44. ^18^F-PSMA-1007 PET/CT (A) failed to detect one lymph node metastasis with maximum diameter of 2,5 mm in histopathological specimens (B), although the lymph node showed intense PSMA staining in immunohistochemistry. Boxed areas in low magnification images are shown in high magnification images. (PNG 3974 kb)

High Resolution Image (TIFF 12096 kb)

Fig. S3 ^18^F-PSMA-1007 PET/CT fusion images of Patient 35. One positive lymph node (short diameter: 6 mm, SUVmax 6.7 g/ml) in the distal iliac region. This was interpreted by both PET readers as malignant, while histopathology results after PLND were negative for lymph node metastases. PSA values dropped <0.006 ng/ml during follow-up after surgery. (PNG 2250 kb)

High Resolution Image (TIF 766 kb)

Fig. S4 Heterogeneity of immunohistochemical PSMA staining in metastatic lymph nodes (A) and aberrant PSMA expression in non-metastatic tissues (B). A: Two examples of metastatic lymph nodes, one with strong and diffuse PSMA staining (upper panel) and the other one with clear heterogeneity in PSMA expression (lower panel). The small separated acinar structures show weak PSMA staining (arrows). B: For some of the patients, weak PSMA staining in lymphoid germinal centres (upper panel) and in endothelial cells of medullary sinuses within lymph nodes (lower panel) were detected. (PNG 8138 kb)

High Resolution Image (TIFF 17915 kb)

## Data Availability

Data are available on request to the corresponding author.
