# Supplementary material for: Prospective comparison of 18F-PSMA-1007 PET/CT, whole-body MRI and CT in primary nodal staging of unfavourable intermediate- and high-risk prostate cancer
Source: Eur J Nucl Med Mol Imaging. 2021 Mar 13;48(9):2951–9. doi: 10.1007/s00259-021-05296-1 (PMC8263440; doi:10.1007/s00259-021-05296-1)
Supplement: Supplementary file 1 — (DOCX 13 kb) [file 259_2021_5296_MOESM1_ESM.docx]

| **Table S1.** Inter-reader agreement in optimistic and pessimistic analysis at the patient level | | |
| --- | --- | --- |
|  | **Kappa (95%CI) optimistic** | **Kappa (95%CI) pessimistic** |
| CT | 0.69 (0.41-0.97) | 0.51 (0.27-0.75) |
| WBMRI with DWI | 0.47 (0.21-0.74) | 0.40 (0.17-0.63) |
| ^18^F-PSMA-1007 PET/CT | 0.89 (0.78-0.99) | 0.86 (0.75-0.98) |
| CT, computed tomography; WBMRI, whole body magnetic resonance imaging; DWI, diffusion-weighted imaging; ^18^F-PSMA-1007 PET/CT, prostate specific membrane antigen positron emission tomography/CT. | | |
