# Supplementary material for: Prospective comparison of 18F-PSMA-1007 PET/CT, whole-body MRI and CT in primary nodal staging of unfavourable intermediate- and high-risk prostate cancer
Source: Eur J Nucl Med Mol Imaging. 2021 Mar 13;48(9):2951–9. doi: 10.1007/s00259-021-05296-1 (PMC8263440; doi:10.1007/s00259-021-05296-1)
Supplement: Supplementary file 2 — (DOCX 18.8 kb) [file 259_2021_5296_MOESM2_ESM.docx]

| **Table S2.** Sensitivity, specificity and accuracy of both readers of each imaging modality in patient-based pessimistic and optimistic analysis in patients who underwent pelvic lymph node dissection. |
| --- |

| **Imaging modality** | **Reader** | **Pessimistic analysis** | | | | | | **Optimistic analysis** | | | |
| --- | --- | --- | --- | --- | --- | --- | --- | --- | --- | --- | --- |
|  |  | **Sensitivity (95%CI)** | | **Specificity (95%CI)** | | **Accuracy (95%CI)** | | **Sensitivity (95%CI)** | | **Specificity (95%CI)** | **Accuracy (95%CI)** |
| CT | 1 | 0.17 (0.03-0.56) | 1.00 (0.74-1.00) | | 0.71 (0.47-0.87) | | 0.00 | | 1.00 (0.74-1.00) | | 0.65 (0.41-0.83) |
|  | 2 | 0.00 | 1.00 (0.74-1.00) | | 0.65 (0.41-0.83) | | 0.00 | | 1.00 (0.74-1.00) | | 0.65 (0.41-0.83) |
|  |  |  |  | |  | |  | |  | |  |
| WBMRI with DWI | 1 | 0.17 (0.03-0.56) | 1.00 (0.74-1.00) | | 0.71 (0.47-0.87) | | 0.00 | | 1.00 (0.74-1.00) | | 0.65 (0.41-0.83) |
|  | 2 | 0.17 (0.03-0.56) | 0.91 (0.62-0.98) | | 0.65 (0.41-0.83) | | 0.17 (0.03-0.56) | | 1.00 (0.74-1.00) | | 0.71 (0.47-0.87) |
|  |  |  |  | |  | |  | |  | |  |
| ^18^F-PSMA-1007 PET/CT | 1 | 0.67 (0.30-0.90) | 0.91 (0.62-0.98) | | 0.82 (0.59-0.94) | | 0.50 (0.19-0.81) | | 0.91 (0.62-0.98) | | 0.76 (0.53-0.90) |
|  | 2 | 0.67 (0.30-0.90) | 0.91 (0.62-0.98) | | 0.82 (0.59-0.94) | | 0.67 (0.30-0.90) | | 0.91 (0.62-0.98) | | 0.82 (0.59-0.94) |

| CT, computed tomography; WBMRI, whole-body magnetic resonance imaging; DWI, diffusion-weighted imaging; ^18^F-PSMA-1007 PET/CT, prostate specific membrane antigen positron emission tomography-CT.   1. Statistically significant difference (p < 0.05) compared to ^18^F-PSMA-1007 PET/CT reader 1 2. Statistically significant difference (p < 0.05) compared to ^18^F-PSMA-1007 PET/CT reader 2 |
| --- |
