# Supplementary material for: Prospective comparison of 18F-PSMA-1007 PET/CT, whole-body MRI and CT in primary nodal staging of unfavourable intermediate- and high-risk prostate cancer
Source: Eur J Nucl Med Mol Imaging. 2021 Mar 13;48(9):2951–9. doi: 10.1007/s00259-021-05296-1 (PMC8263440; doi:10.1007/s00259-021-05296-1)
Supplement: Supplementary file 3 — (DOCX 14 kb) [file 259_2021_5296_MOESM3_ESM.docx]

**Table S3.** The total number of reported lesions by both readers of each imaging modality and their concordance

with the reference standard diagnosis at lesion level in patients that underwent pelvic lymph node dissection ^a^.

| **Imaging modality** | **Reader** | **Number of positive lesions reported** | **Number of true positive lesions** | **Detection rate of true positive lesions** | **Number of false positive lesions** | **Number of false negative lesions** | **Number of equivocal lesions reported** |
| --- | --- | --- | --- | --- | --- | --- | --- |
| CT | 1 | 0 | 0 | 0% | 0 | 15 | 1 |
|  | 2 | 0 | 0 | 0% | 0 | 15 | 0 |
|  |  |  |  |  |  |  |  |
| WBMRI with DWI | 1 | 0 | 0 | 0% | 0 | 15 | 1 |
|  | 2 | 2 | 1 | 7% | 1 | 14 | 0 |
|  |  |  |  |  |  |  |  |
| ^18^F-PSMA-1007 PET/CT | 1 | 4 | 3 | 20% | 1 | 12 | 2 |
|  | 2 | 7 | 5 | 27% | 2 | 10 | 0 |

CT, computed tomography; WBMRI, whole-body magnetic resonance imaging; DWI, diffusion-weighted imaging; ^18^F-PSMA-1007 PET/CT, prostate specific membrane antigen positron emission tomography-CT.

^a^ There were 15 metastatic lymph nodes according to reference standard diagnosis. 14 lymph nodes were confirmed by histology (11 by haematoxylin and eosin, 3 only by immunohistochemistry). One lymph node positive in ^18^F-PSMA-1007 PET-CT was located in the perirectal region.
