# Supplementary material for: Prospective comparison of 18F-PSMA-1007 PET/CT, whole-body MRI and CT in primary nodal staging of unfavourable intermediate- and high-risk prostate cancer
Source: Eur J Nucl Med Mol Imaging. 2021 Mar 13;48(9):2951–9. doi: 10.1007/s00259-021-05296-1 (PMC8263440; doi:10.1007/s00259-021-05296-1)
Supplement: Supplementary file 4 — (DOCX 17 kb) [file 259_2021_5296_MOESM4_ESM.docx]

**Table S4.** Number of lymph node metastases detected in H&E and IHC from pelvic lymph node dissection specimens and number of lymph node reported as malignant on ^18^F-PSMA-1007 PET/CT by reader 1 and 2.

| Patient number | Number of LN metastases in H&E | Number of LN metastases in pan-cytokeratin and PSMA IHC | Longest diameter of the LN metastasis (mm) | Number of malignant LN reported on ^18^F-PSMA-1007 PET/CT (reader 1) | Number of malignant LN reported on ^18^F-PSMA-1007 PET/CT (reader 2) | Highest SUVmax | LN size on PET/CT  (short axis, mm) |
| --- | --- | --- | --- | --- | --- | --- | --- |
| 12 | 1 | 2 | 13, 1^b^ | 1 | 1 | 15.2 | 4 |
| 30 | 1 | 1 | 4 | 0 | 0 | - | - |
| 44 | 1 | 1 | 2.5 | 0 | 0 | - | - |
| 48 | 5 | 6 | 8.5, 7, 4, 4, 1, 0.1^b^ | 1 | 1 | 8.1 | 5 |
| 78 | 3 | 4 | 5.5, 1.5, 0.6, 0.5^b^ | 0 | 1 | 6.1 | 7 |
| 22^a^ | 0 | 0 | - | 1 | 1 | 9.2 | 6 |
| 35 | 0 | 0 | - | 1 | 2 | 6.7, 4.0 | 5, 8 |
| 7 | 0 | 0 | - | 0 | 0 | - |  |
| 8 | 0 | 0 | - | 0 | 0 | - |  |
| 13 | 0 | 0 | - | 0 | 0 | - |  |
| 15 | 0 | 0 | - | 0 | 0 | - |  |
| 43 | 0 | 0 | - | 0 | 0 | - |  |
| 45 | 0 | 0 | - | 0 | 0 | - |  |
| 56 | 0 | 0 | - | 0 | 0 | - |  |
| 65 | 0 | 0 | - | 0 | 0 | - |  |
| 0 | 0 | 0 | - | 0 | 0 | - |  |
| 71 | 0 | 0 | - | 0 | 0 | - |  |

^18^F-PSMA-1007 PET/CT, prostate specific membrane antigen positron emission tomography/computed tomography; H&E, haematoxylin and eosin; IHC, immunohistochemistry; LN, lymph node

^a^ Patient presented with ^18^F-PSMA-1007 PET-positive lymph node in the perirectal region, therefore not removed during pelvic lymph node dissection

^b^ Lymph nodes (n=3) detected only in IHC
