# Supplementary material for: Prospective comparison of 18F-PSMA-1007 PET/CT, whole-body MRI and CT in primary nodal staging of unfavourable intermediate- and high-risk prostate cancer
Source: Eur J Nucl Med Mol Imaging. 2021 Mar 13;48(9):2951–9. doi: 10.1007/s00259-021-05296-1 (PMC8263440; doi:10.1007/s00259-021-05296-1)
Supplement: Supplementary file 5 — (DOCX 14 kb) [file 259_2021_5296_MOESM5_ESM.docx]

**Table S5**. Overview of PSMA PET/CT sensitivity and specificity in primary pelvic nodal staging from prospective studies that used histopathology as a reference standard.

| First author (year) [ref] | Study design | Tracer | Patients in the study (patients with histological verification) | Patients with LN metastases | Sensitivity  patient-based | Specificity  patient-based |
| --- | --- | --- | --- | --- | --- | --- |
| van Leeuwen (2017) [17] | prospective | ^68^Ga-PSMA-11 | 30 (30) | 11 | 0.64 | 0.95 |
| Petersen (2019) [22] | prospective | ^68^Ga-PSMA-11 | 20 (20) | 5 | 0.39 | 0.1 |
| van Kalmthout (2020) [19] | prospective | ^68^Ga-PSMA-11 | 103 (97) | 41 | 0.42 | 0.91 |
| Jansen (2020) [12] | prospective | ^18^F-DCFPyL | 117 (117) | 17 | 0.41 | 0.94 |
