# Supplementary figures and images for: Prospective comparison of 18F-PSMA-1007 PET/CT, whole-body MRI and CT in primary nodal staging of unfavourable intermediate- and high-risk prostate cancer
Source: Eur J Nucl Med Mol Imaging. 2021 Mar 13;48(9):2951–9. doi: 10.1007/s00259-021-05296-1 (PMC8263440; doi:10.1007/s00259-021-05296-1)

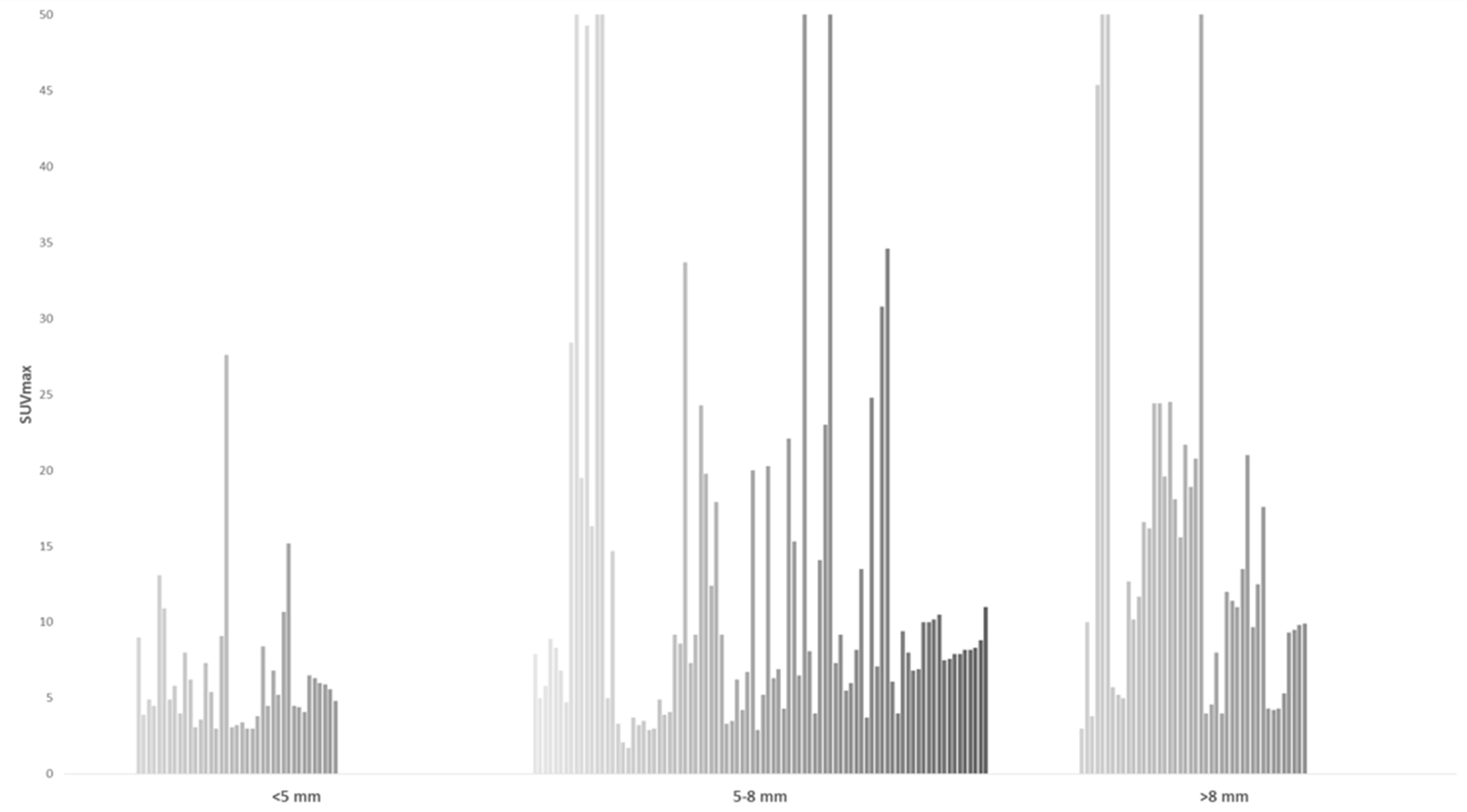

Supplement: Supplementary file 6 — SUVmax values of metastatic lymph nodes (reference standard) divided according to lymph node short diameter. Analysis of Reader 1 (higher detection rate) is presented. (PNG 116 kb) [file 259_2021_5296_Fig2_ESM.png]

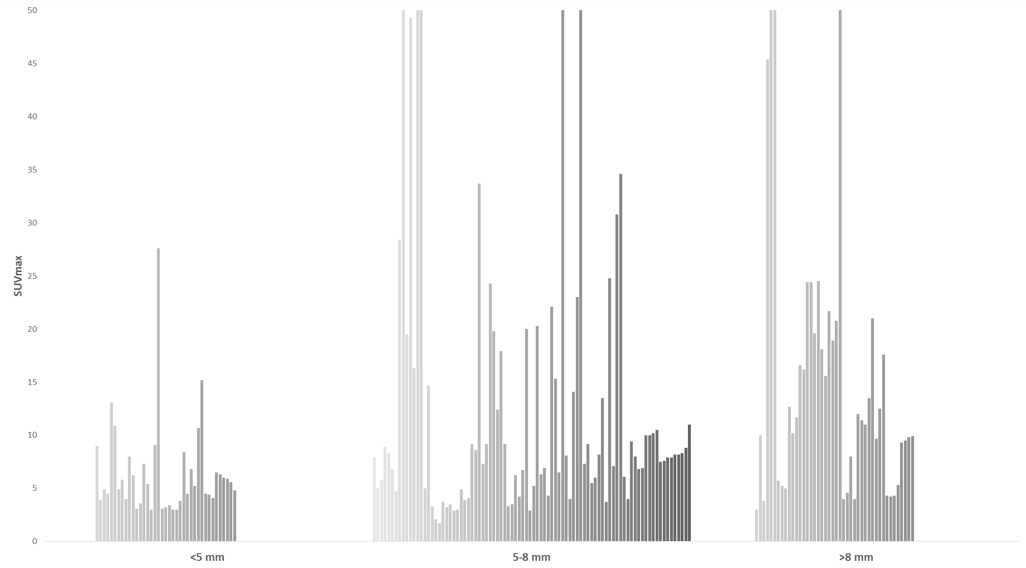

Supplement: Supplementary file 7 — High Resolution Image (TIFF 164 kb) [file 259_2021_5296_MOESM6_ESM.tiff]

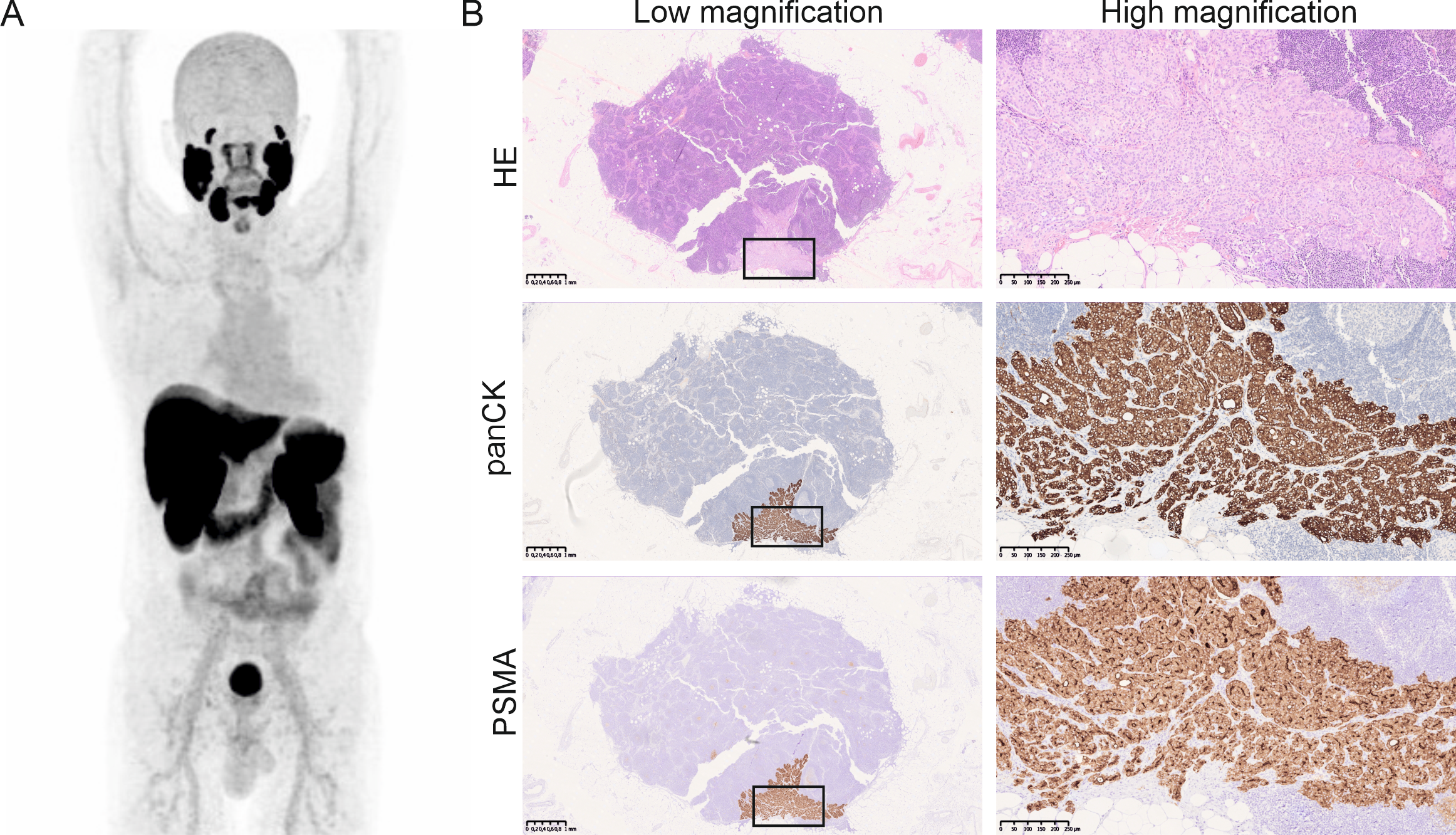

Supplement: Supplementary file 8 — Imaging and histopathological findings of Patient 44. 18F-PSMA-1007 PET/CT (A) failed to detect one lymph node metastasis with maximum diameter of 2,5 mm in histopathological specimens (B), although the lymph node showed intense PSMA staining in immunohistochemistry. Boxed areas in low magnification images are shown in high magnification images. (PNG 3974 kb) [file 259_2021_5296_Fig3_ESM.png]

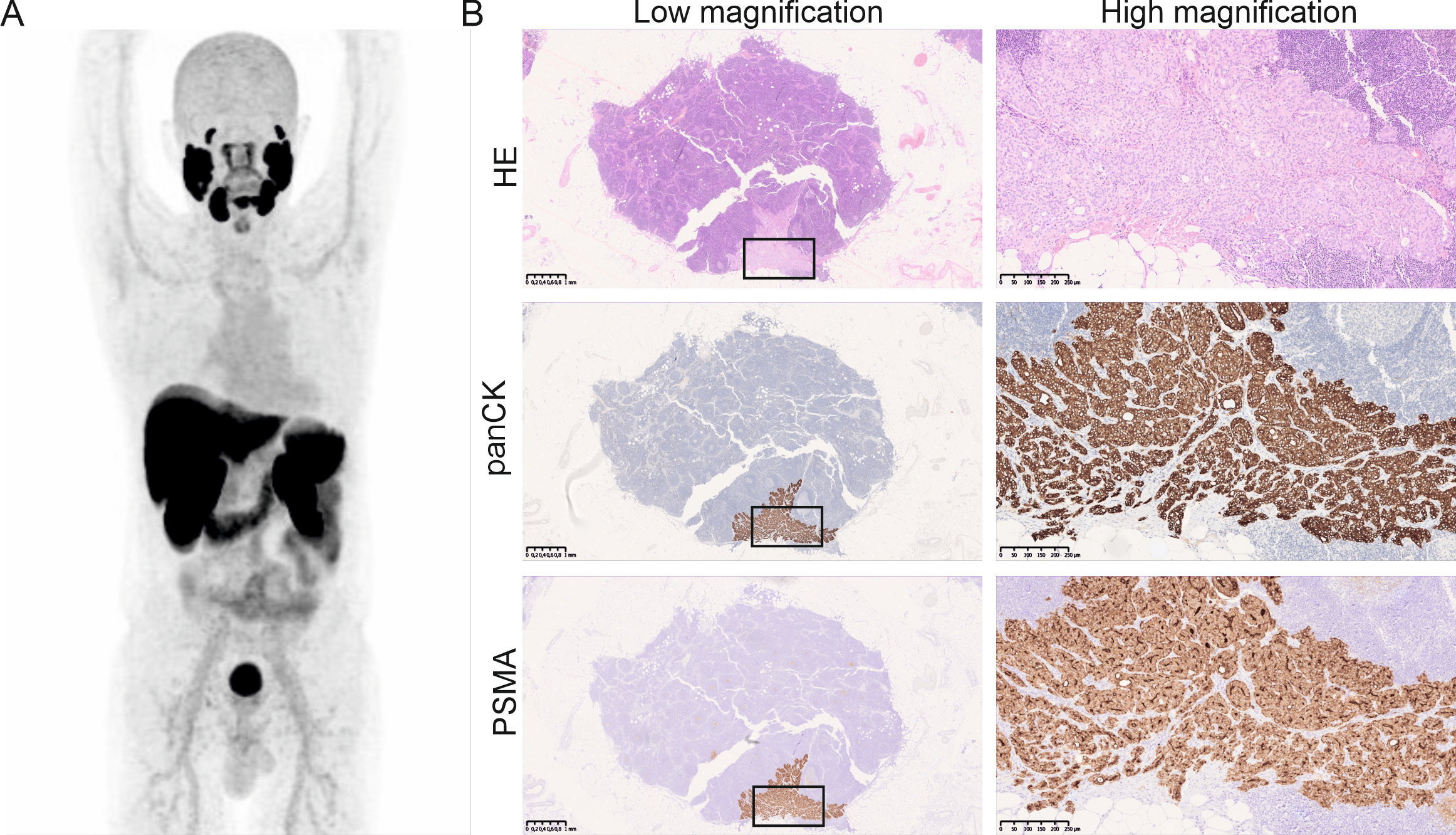

Supplement: Supplementary file 9 — High Resolution Image (TIFF 12096 kb) [file 259_2021_5296_MOESM7_ESM.tiff]

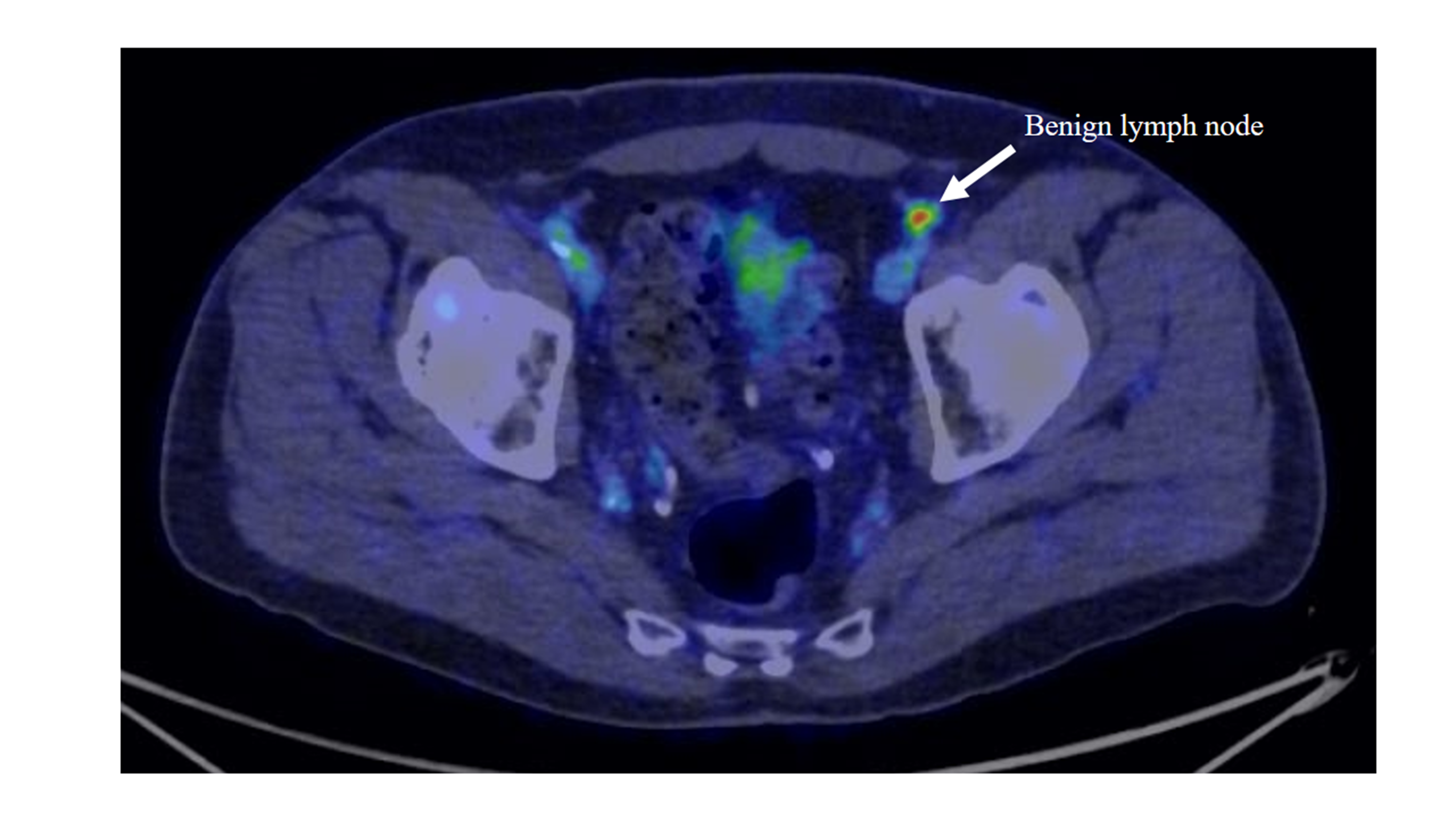

Supplement: Supplementary file 10 — 18F-PSMA-1007 PET/CT fusion images of Patient 35. One positive lymph node (short diameter: 6 mm, SUVmax 6.7 g/ml) in the distal iliac region. This was interpreted by both PET readers as malignant, while histopathology results after PLND were negative for lymph node metastases. PSA values dropped <0.006 ng/ml during follow-up after surgery. (PNG 2250 kb) [file 259_2021_5296_Fig4_ESM.png]

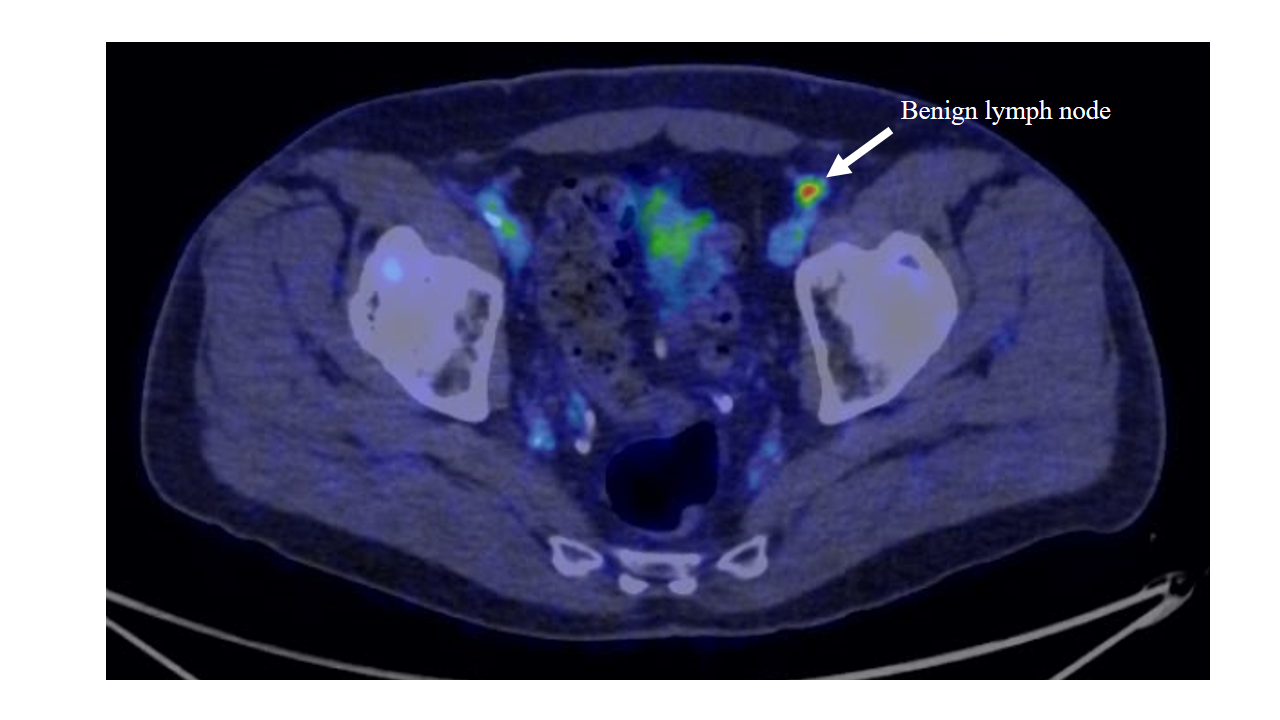

Supplement: Supplementary file 11 — High Resolution Image (TIF 766 kb) [file 259_2021_5296_MOESM8_ESM.tif]

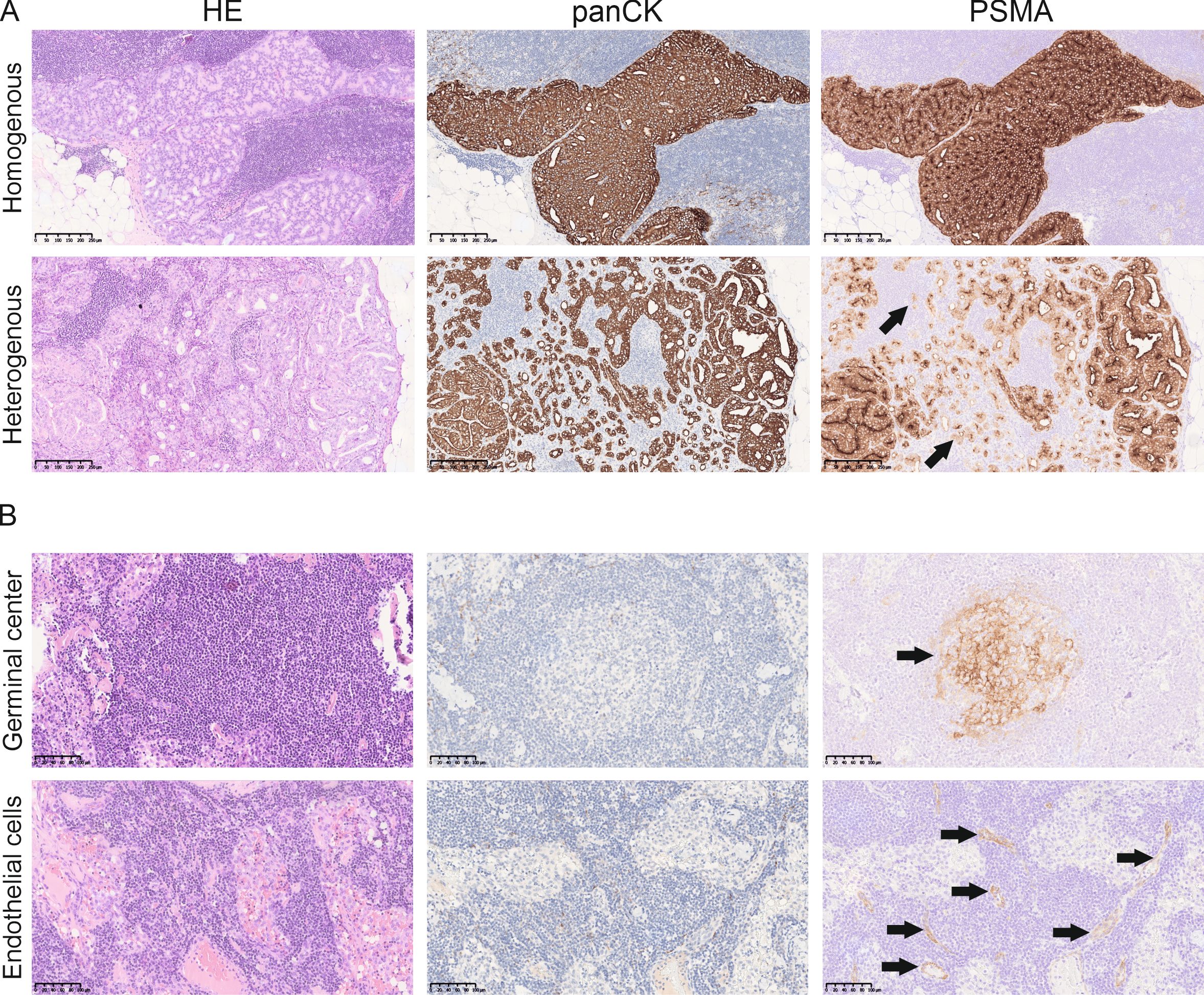

Supplement: Supplementary file 12 — Heterogeneity of immunohistochemical PSMA staining in metastatic lymph nodes (A) and aberrant PSMA expression in non-metastatic tissues (B). A: Two examples of metastatic lymph nodes, one with strong and diffuse PSMA staining (upper panel) and the other one with clear heterogeneity in PSMA expression (lower panel). The small separated acinar structures show weak PSMA staining (arrows). B: For some of the patients, weak PSMA staining in lymphoid germinal centres (upper panel) and in endothelial cells of medullary sinuses within lymph nodes (lower panel) were detected. (PNG 8138 kb) [file 259_2021_5296_Fig5_ESM.png]

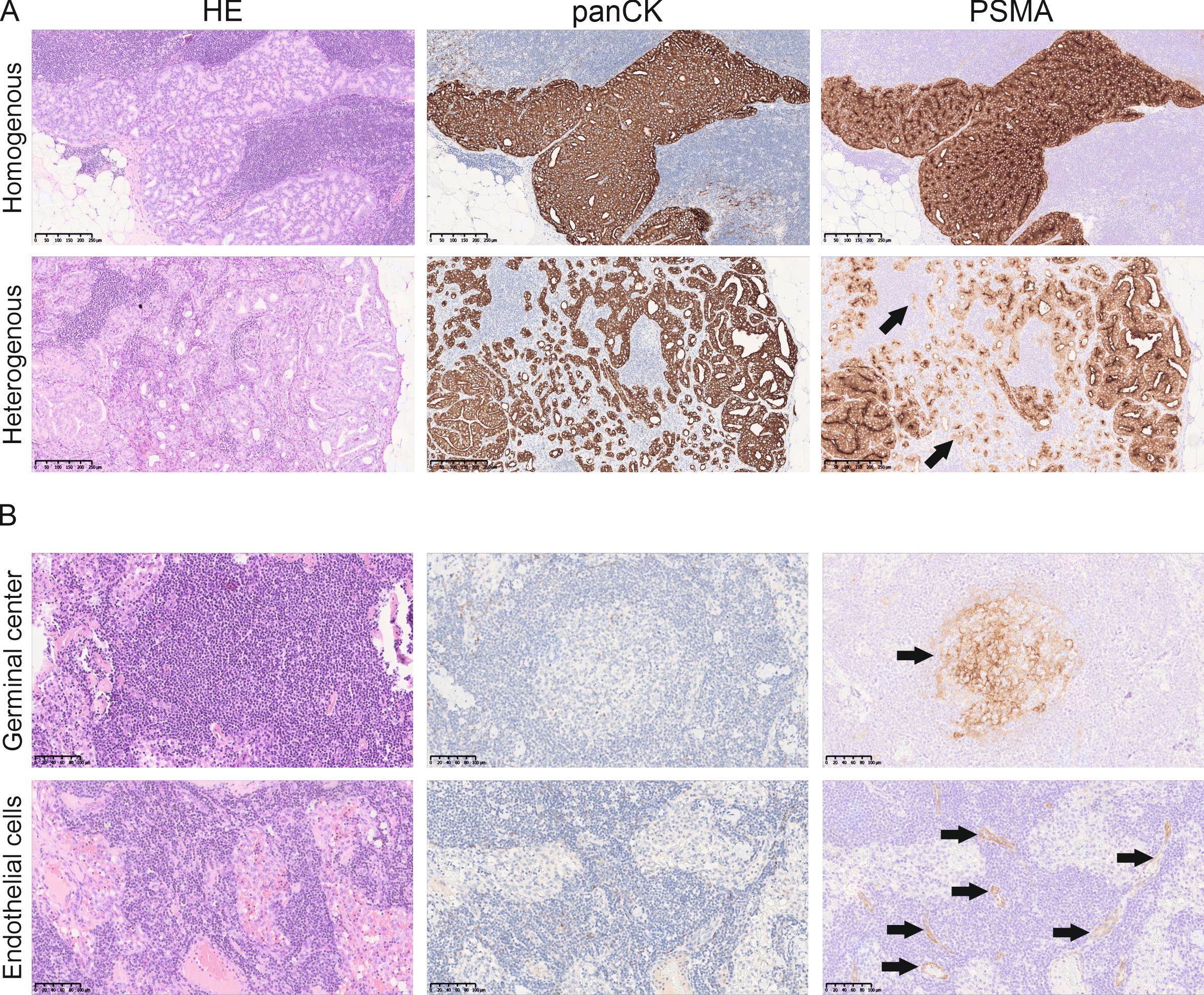

Supplement: Supplementary file 13 — High Resolution Image (TIFF 17915 kb) [file 259_2021_5296_MOESM9_ESM.tiff]
